# The emerging role of 4D flow MRI in abdominal interventional radiology

**DOI:** 10.1007/s00261-026-05526-w

**Published:** 2026-04-25

**Authors:** Ryota Hyodo, Ryota Horiguchi, Mami Iima, Shinji Naganawa

**Affiliations:** 1 Department of Radiology, Nagoya University, Nagoya, Japan; 2 Department of Fundamental Development for Advanced Low Invasive Diagnostic Imaging, Nagoya University, Nagoya, Japan

**Keywords:** 4D Flow MRI, Interventional radiology, Hemodynamics, Portal vein embolization, Endovascular aortic repair

## Abstract

Four-dimensional flow magnetic resonance imaging (4D Flow MRI) enables time-resolved three-dimensional visualization and retrospective quantification of vascular hemodynamics within a single volumetric acquisition. Beyond morphology-based imaging, it provides comprehensive assessment of multidirectional flow patterns, collateral redistribution, and treatment-related hemodynamic changes across vascular territories. In abdominal interventional radiology (IR), 4D Flow MRI may offer incremental value in clinical scenarios where morphology alone is insufficient to characterize physiologically relevant hemodynamics. This review summarizes its applications in aortic interventions, portal venous interventions, and hepatic venous outflow disorders. In these settings, 4D Flow MRI may support functional assessment of endoleaks, evaluation of portal flow redistribution, and characterization of venous outflow obstruction and treatment response. Among current applications, endoleak characterization and early assessment of portal hemodynamic change after portal vein embolization, which may reflect subsequent liver hypertrophy, represent particularly promising use cases. However, current evidence remains limited and largely observational. Important technical considerations, including velocity encoding strategy, physiologic variability, metal-related artifacts, and workflow integration, must be addressed. In practical terms, 4D Flow MRI is best regarded as a complementary or problem-solving technique rather than a modality for routine clinical decision-making. Further multicenter validation and outcome-driven studies are required to define its clinical impact in IR practice.

## Introduction

**Fig. 1:**
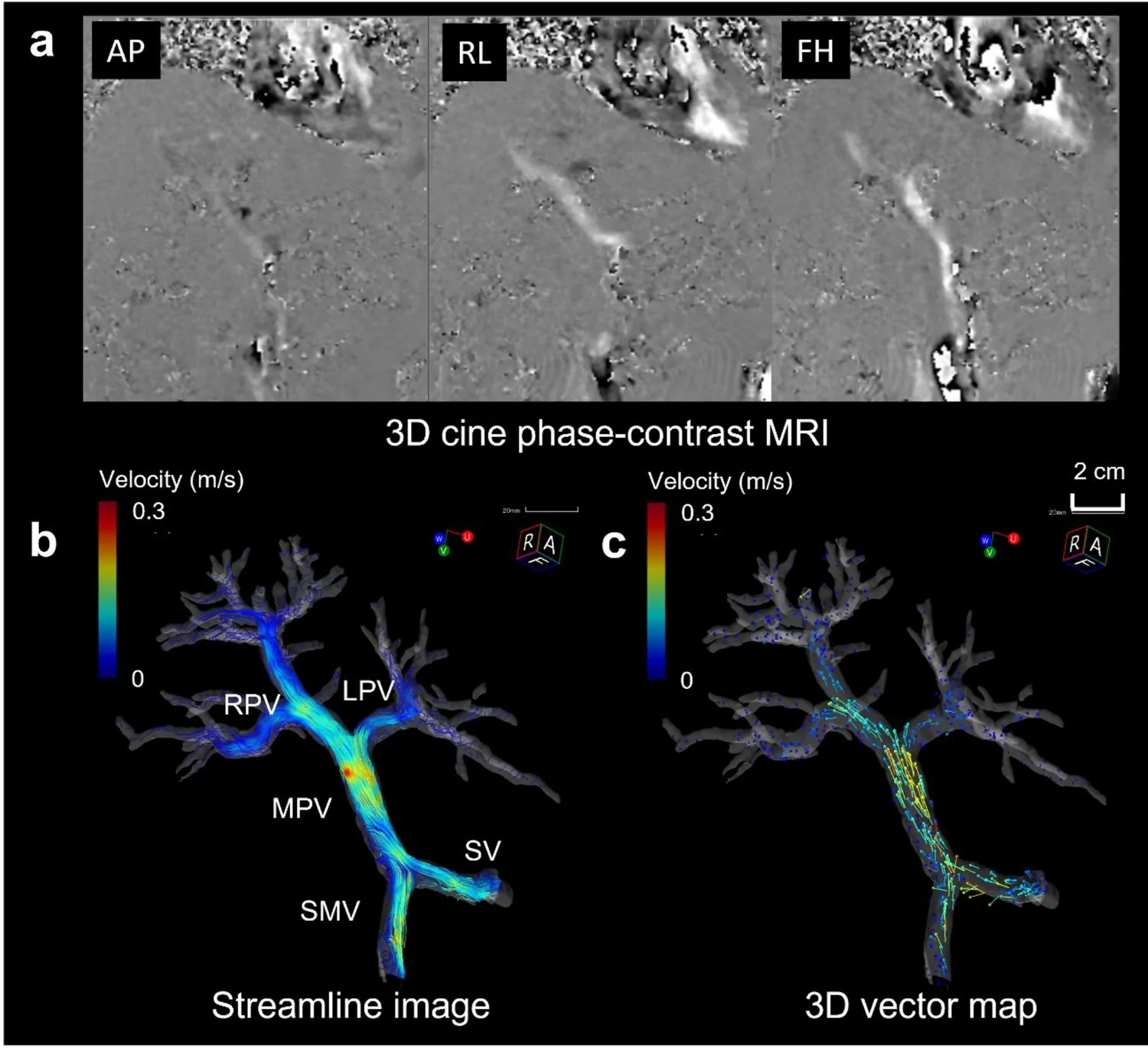
Principles of 4D Flow MRI for visualization and quantification of vascular hemodynamics. Representative case (female, 70 s). **a** Velocity-encoded phase images represent velocity components in three orthogonal directions: anterior–posterior (AP), right–left (RL), and foot–head (FH). These components are combined to reconstruct the three-dimensional velocity vector field **b** Streamline visualization derived from the instantaneous velocity field illustrates spatial flow pathways at a given cardiac phase by connecting local velocity vectors, enabling intuitive assessment of flow patterns **c** Three-dimensional vector maps depict local flow direction and velocity magnitude within segmented vessels. Velocity magnitude is color-coded in meters per second. *MPV* Main portal vein, *SMV* Superior mesenteric vein, *SV* Splenic vein, *LPV* Left portal vein, *RPV* Right portal vein

Four-dimensional flow magnetic resonance imaging (4D Flow MRI) enables time-resolved, three-dimensional visualization and quantification of blood flow throughout the cardiac cycle [1]. By acquiring volumetric phase-contrast data, it provides velocity information across an entire vascular territory within a single examination, allowing retrospective analysis of flow direction, velocity, and volumetric flow at multiple locations (Fig. 1). From these data, flow visualization techniques such as streamline and pathline can be generated. Streamlines depict instantaneous flow patterns by tracing curves tangent to the velocity field at a given time point, whereas pathlines represent the trajectories of virtual particles over time. Initially developed for large thoracic vessels, such as the aorta and pulmonary arteries, its application has expanded to smaller-caliber and lower-velocity vascular systems, including the abdominal visceral arteries, portal venous system, and systemic veins [2, 3]. Recent consensus statements have summarized current technical considerations and recommended approaches for acquisition and analysis [4, 5].

Interventional radiology (IR) has become an essential minimally invasive treatment modality across a wide range of vascular diseases. However, both treatment planning and post-procedural evaluation continue to rely heavily on morphology-based imaging. Computed tomography (CT) and conventional MRI provide excellent spatial resolution and global anatomical assessment, but they do not allow direct quantification of blood flow. Two-dimensional phase-contrast MRI enables flow measurement but requires prospective plane selection and does not provide comprehensive volumetric assessment. Ultrasound is widely available but is operator-dependent and often limited by acoustic window constraints [6]. Catheter angiography offers high temporal resolution but is invasive and does not readily provide quantitative flow information [7].

In abdominal IR, clinically relevant questions often extend beyond anatomical findings alone. For example, it may be necessary to determine whether portal venous flow has been adequately redistributed after embolization, whether an apparent vascular narrowing is hemodynamically significant, or whether persistent enhancement after endovascular aortic repair reflects high-pressure inflow or low-flow collateral circulation. In such situations, morphology-based imaging may identify abnormalities but may not fully characterize their physiologic significance. In contrast, 4D Flow MRI enables comprehensive assessment of flow direction, redistribution, and quantitative hemodynamics within a single acquisition, which may support more precise treatment planning and objective evaluation of therapeutic response.

**Fig. 2:**
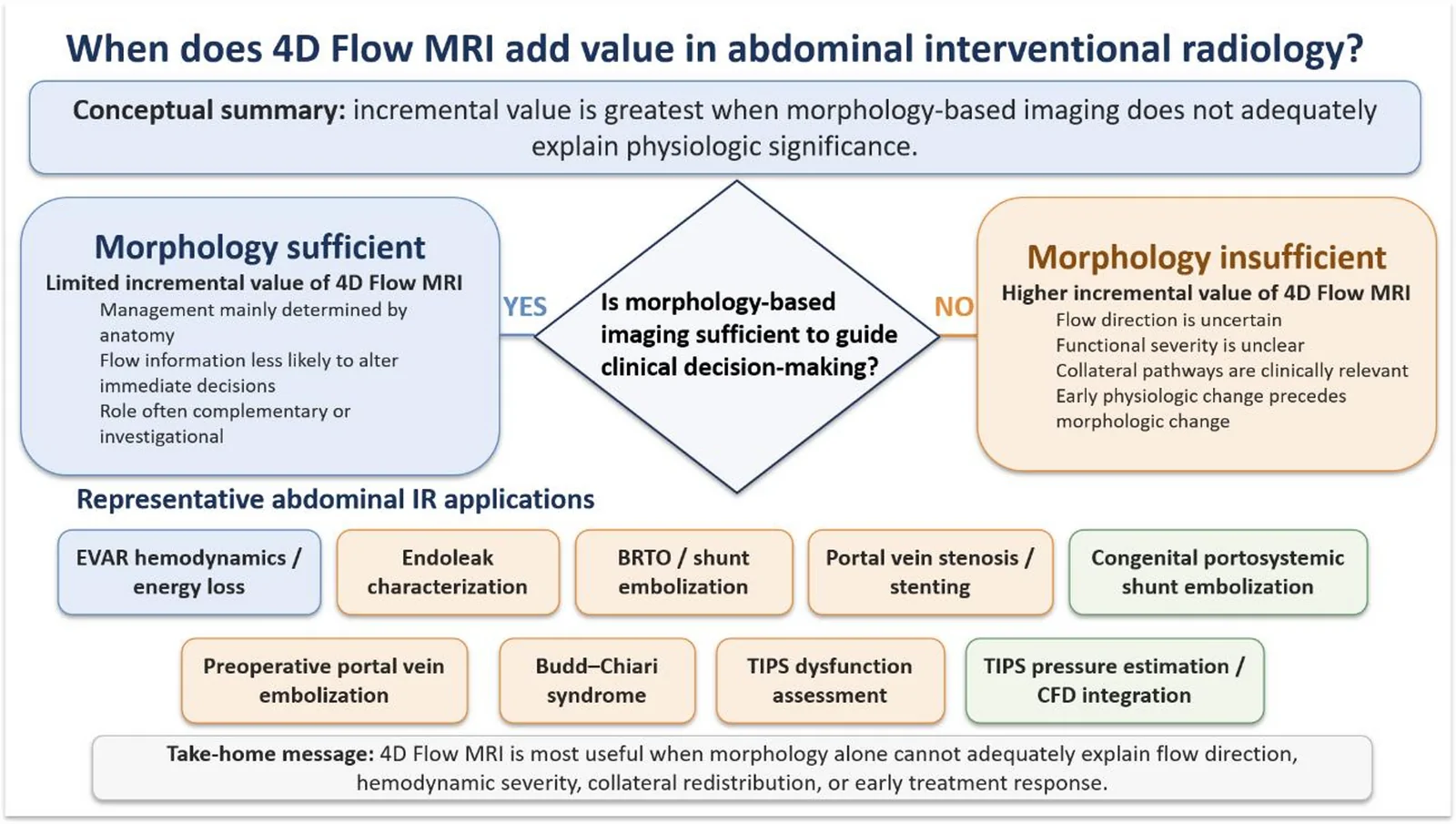
Conceptual framework for the incremental value of 4D Flow MRI in abdominal interventional radiology

A useful conceptual framework for understanding the role of 4D Flow MRI in abdominal IR is whether morphology-based imaging alone is sufficient to explain clinically relevant hemodynamics. When vascular morphology adequately reflects physiologic conditions, the incremental value of 4D Flow MRI is often limited and primarily complementary. In contrast, when morphology is insufficient to characterize flow direction, functional severity, collateral pathways, or early physiologic change after intervention, 4D Flow MRI may provide higher incremental value. This conceptual framework is illustrated in Fig. 2.

In this review, we summarize representative clinical applications of 4D Flow MRI in abdominal IR and discuss its incremental clinical value, technical considerations, current evidence, and practical limitations for clinical implementation.

The incremental value of 4D Flow MRI depends on whether morphology-based imaging alone is sufficient to explain the physiologic significance of vascular disease or treatment response. When anatomy and morphology adequately define management, the additional role of 4D Flow MRI is often limited and complementary. In contrast, when morphology alone is insufficient to characterize flow direction, functional severity, collateral hemodynamics, or early post-treatment physiologic change, 4D Flow MRI may provide higher added value. Representative abdominal IR applications are positioned within this framework, including endoleak characterization after EVAR, portal venous interventions, Budd–Chiari syndrome, and TIPS assessment.

## Clinical scenarios in interventional radiology

### Aortic interventions

#### Hemodynamic assessment after endovascular aortic repair (EVAR)

Abdominal aortic aneurysm (AAA) remains a significant cause of mortality; ruptured AAA is frequently fatal and is associated with high overall mortality rates [8, 9]. Both surgical repair and endovascular treatment are indicated for rupture prevention and acute management. In contemporary practice, EVAR has become the first-line treatment for most patients with suitable anatomy [9]. Preprocedural planning relies primarily on dynamic contrast-enhanced CT (DCE-CT) to assess aneurysm morphology, branch vessel relationships, and landing zone characteristics. After EVAR, surveillance focuses on aneurysm sac size, device integrity, and the presence of endoleaks.

**Fig. 3:**
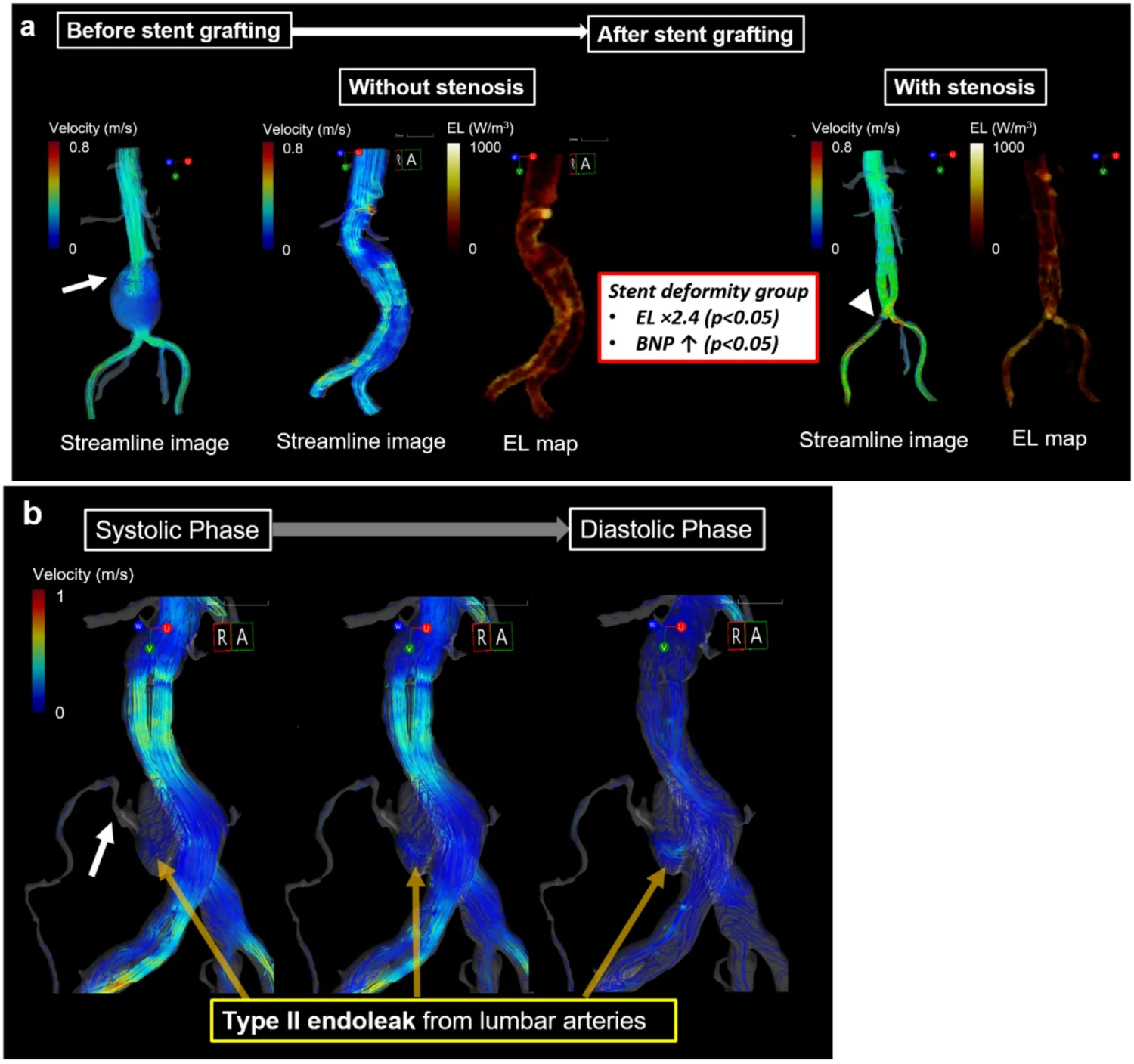
4D Flow MRI for hemodynamic assessment of abdominal aortic aneurysm and endovascular aneurysm repair (EVAR)-related complications. **a** Comparison of two representative cases (female, 80 s; male, 70 s). Hemodynamic evaluation before and after EVAR. Before stent grafting, streamline visualization demonstrates vortex flow within the abdominal aortic aneurysm sac (arrow) with reduced velocity, reflecting disturbed flow. After successful stent grafting without stenosis, intra-aneurysmal flow signals are no longer apparent and laminar flow is restored within the stent graft lumen. In contrast, in a case with stent graft limb deformity, focal stenosis (arrowhead) is associated with increased local energy loss (EL) at the narrowed segment. Increased EL may reflect impaired hemodynamic efficiency beyond morphologic assessment. Adapted from Horiguchi R et al. J Magn Reson Imaging 2023, licensed under CC BY-NC. **b** Representative case (male, 70 s). Evaluation of type II endoleak after EVAR. Streamline visualization demonstrates retrograde inflow from a lumbar artery into the aneurysm sac, consistent with a type II endoleak (arrows). Flow within the sac is particularly evident during diastole, highlighting its dynamic nature. Compared with conventional imaging, 4D Flow MRI allows visualization of flow direction and temporal flow patterns adjacent to the stent graft, supporting functional assessment of endoleaks

Compared with DCE-CT, which provides morphologic assessment, 4D Flow MRI offers complementary hemodynamic information within the same vascular territory. Streamline visualization enables intuitive assessment of flow organization and depicts complex vortical flow patterns within the aneurysm sac (Fig. 3a). Quantitative analysis further characterizes local flow behavior. After EVAR, elimination of intra-aneurysmal recirculation and restoration of luminal continuity alter aortic hemodynamics, including changes in velocity distribution and energy dissipation. In particular, local energy loss (EL) has been reported to increase after EVAR and may be locally elevated in cases of stent graft limb deformity or focal stenosis (Fig. 3a) [2, 10].

These observations suggest that 4D Flow MRI can identify device-related flow inefficiency that is not apparent on morphology alone. However, the clinical significance of such findings remains uncertain. Although associations between altered hemodynamics and biomarkers such as brain natriuretic peptide (BNP) have been reported, these findings are preliminary and have not been linked to clinical outcomes or management strategies [2]. In practical terms, 4D Flow MRI may be considered in selected cases with suspected device-related flow disturbance or unexplained symptoms despite unremarkable morphologic findings. However, its role in influencing reintervention thresholds or surveillance strategies has not been established, and routine use in post-EVAR follow-up is not currently supported.

#### Assessment of endoleaks after EVAR

Aneurysm sac enlargement and late rupture remain clinically significant complications after stent graft placement. Accordingly, routine surveillance includes serial evaluation of aneurysm diameter and contrast-enhanced imaging for endoleak detection [11]. Endoleaks are broadly classified into high-pressure types, associated with direct communication between the aortic lumen and the aneurysm sac, and low-pressure types resulting from retrograde collateral inflow [12]. Because management differs substantially between these categories, accurate classification is essential for risk stratification and treatment planning. Although catheter angiography remains the reference standard, it is invasive. DCE-CT reliably detects endoleaks but does not provide direct information on flow direction, and differentiation between high-pressure inflow (type I/III) and low-flow collateral filling (type II) may be challenging in selected cases.

4D Flow MRI enables direct visualization of intra-sac flow and allows assessment of flow direction using vector analysis (Fig. 3b) [13]. This information is not available from CT alone and may help distinguish clinically relevant high-pressure inflow from low-flow retrograde perfusion. Prior studies suggest that 4D Flow MRI may improve endoleak detection and classification compared with CT angiography [13]. However, this application is technically challenging because of the wide velocity range between aortic flow and slow endoleak components, making appropriate velocity encoding critical. Multi-VENC approaches may improve dynamic range and facilitate simultaneous assessment of high- and low-velocity flow components [4, 5, 14, 15], but require optimized acquisition and remain limited in availability.

In this context, 4D Flow MRI may provide incremental value as a noninvasive adjunct for functional characterization of endoleaks, particularly in cases with equivocal findings on conventional imaging or discordance between sac behavior and CT findings. However, current evidence is limited, and it remains unclear whether such hemodynamic information should influence reintervention thresholds or alter surveillance strategies. Accordingly, 4D Flow MRI is best considered a complementary, problem-solving tool rather than a modality for routine post-EVAR follow-up.

### Portal venous interventions

Portal venous interventions represent a distinct hemodynamic environment characterized by low-velocity, multidirectional flow, and frequent collateralization. In this setting, treatment response is often determined not only by morphologic change but also by changes in portal flow distribution and collateral hemodynamics.

#### Balloon-occluded retrograde transvenous obliteration for gastric varices or hepatic encephalopathy

In advanced chronic liver disease, progressive hepatic fibrosis increases intrahepatic vascular resistance, leading to portal hypertension and the development of hepatofugal collateral pathways, commonly referred to as portosystemic shunts (PSS) [16, 17]. These shunts may result in life-threatening variceal bleeding or hepatic encephalopathy due to diversion of portal blood away from the liver. Balloon-occluded retrograde transvenous obliteration (BRTO) is an established endovascular technique that occludes the draining vein of a PSS to eliminate the shunt and redirect portal venous flow toward the liver [18–21].

**Fig. 4:**
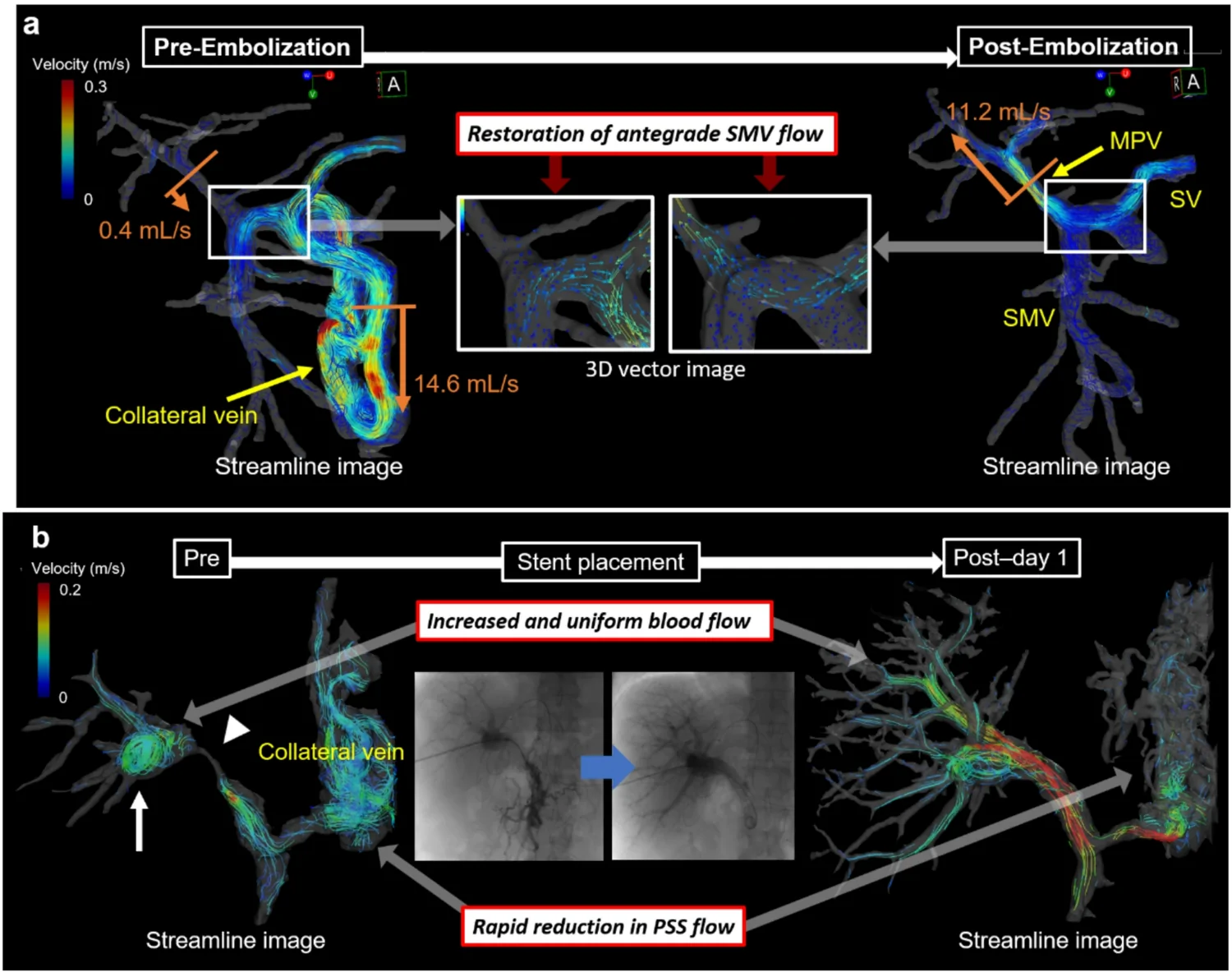
Hemodynamic redistribution after portal venous interventions assessed by 4D Flow MRI **a** Representative case (female, 70 s). Flow restoration after balloon-occluded retrograde transvenous obliteration (BRTO). Pre-embolization streamline and vector visualizations demonstrate a large portosystemic shunt arising from a dilated inferior mesenteric vein, diverting mesenteric venous return into the systemic circulation. Three-dimensional vector analysis shows near-complete diversion of superior mesenteric vein (SMV) flow through the shunt and hepatofugal flow in the main portal vein (MPV). After shunt embolization, antegrade flow in the SMV and splenic vein (SV) is restored, indicating redirection of portal flow toward the liver. Adapted from Hyodo R et al. Radiologia Medica 2022, licensed under CC BY 4.0. **b** Representative case (female, 30 s). Flow normalization after portal vein stent placement. Pre-interventional streamline visualization demonstrates severe MPV stenosis with post-stenotic dilatation, vortex formation, heterogeneous intrahepatic portal perfusion, and prominent portosystemic shunt (PSS) flow. Pre- and post-interventional angiography confirms severe MPV stenosis and restoration of portal vein patency after stent placement. Following percutaneous transhepatic portal vein stenting, postoperative 4D Flow MRI demonstrates increased flow velocity and more homogeneous intrahepatic flow distribution. Although PSS morphology remains unchanged, shunt flow is markedly reduced, indicating early hemodynamic improvement. Adapted from Hyodo R et al. Magn Reson Med Sci 2021, licensed under CC BY-NC-ND 4.0

Compared with CT or conventional angiography, which primarily depict shunt anatomy, 4D Flow MRI enables three-dimensional and quantitative assessment of global portal hemodynamics before and after BRTO. In patients with hepatic encephalopathy, pre-treatment imaging often demonstrates substantial diversion of portal inflow through the shunt, with reduced flow in the main portal vein (MPV) and, in some cases, retrograde superior mesenteric vein (SMV) flow (Fig. 4a). Following embolization, 4D Flow MRI can demonstrate restoration of hepatopetal flow and increased portal inflow, providing a direct visualization of treatment-induced hemodynamic redistribution. Such information may support physiologic confirmation of treatment response in cases of PSS-related encephalopathy [22]. In contrast, in BRTO performed for variceal bleeding, the primary clinical objective is elimination of the bleeding source, and the added value of detailed hemodynamic assessment is less clear. Although post-treatment changes in shunt flow can be visualized, the impact of these findings on clinical management remains uncertain. Similarly, while preprocedural identification of prominent shunts or reversed portal flow may provide additional physiologic context [7], its role in determining treatment indication has not been established.

In practical terms, 4D Flow MRI may be most useful in selected cases where the physiologic significance of a portosystemic shunt is unclear or when objective confirmation of post-treatment hemodynamic response is clinically relevant, particularly in patients with hepatic encephalopathy. However, its role in guiding treatment indication or predicting clinical outcomes remains investigational, and routine use in all BRTO cases is not currently supported.

#### Portal vein stenting for portal vein stenosis after surgery

Portal vein stenosis may occur after hepatobiliary surgery requiring portal vein resection and reconstruction, including major hepatectomy or liver transplantation [23–25]. In addition, extrinsic compression or tumor invasion from pancreatic or biliary malignancies may result in progressive portal vein narrowing. These conditions can reduce intrahepatic portal inflow and promote development of hepatofugal collateral pathways, potentially leading to hepatic dysfunction, ascites, and gastroesophageal varices. Portal vein stent placement aims to restore physiologic hepatopetal portal flow and alleviate complications associated with portal hypertension and collateral circulation [26–28]. However, conventional imaging modalities such as DCE-CT and ultrasound have limited ability to comprehensively assess the hemodynamic significance of portal vein narrowing, and even catheter angiography may not adequately depict territories with reduced perfusion.

4D Flow MRI provides time-resolved volumetric assessment of the entire portal venous system, enabling simultaneous visualization and quantitative evaluation of stenosis-related flow disturbance, intrahepatic perfusion heterogeneity, and collateral pathway development within a single acquisition (Fig. 4b) [29]. Hemodynamic impairment may be substantial even when morphologic stenosis appears mild on conventional imaging, and identification of marked flow imbalance or predominant hepatofugal collateral flow may provide additional physiologic information in cases where morphology alone is inconclusive. Following stent placement, 4D Flow MRI can demonstrate improved intrahepatic flow distribution and reduction of collateral flow, allowing early assessment of hemodynamic response before measurable morphologic changes become apparent.

In this context, 4D Flow MRI may provide incremental value in selected cases by helping to clarify the functional significance of portal vein stenosis when conventional imaging findings are equivocal, or by providing early physiologic confirmation of treatment response after stent placement. However, current evidence remains limited, and no standardized hemodynamic thresholds have been established to guide intervention. Accordingly, while 4D Flow MRI may support clinical interpretation in challenging cases, its role in determining treatment indications or defining intervention thresholds has not been established, and routine use in all patients with portal vein stenosis is not currently supported.

#### Embolization for congenital portal abnormality

Congenital abnormalities of the portal venous system encompass a spectrum of vascular malformations, including large PSS that divert portal flow directly into the systemic circulation [30, 31]. In patients with substantial shunt flow, a large proportion of portal venous inflow bypasses the liver, resulting in reduced hepatopetal perfusion and compensatory arterialization of hepatic blood supply. Chronic portal deprivation may lead to hepatic parenchymal atrophy, development of focal nodular hyperplasia or hepatocellular adenoma-like lesions, and hyperammonemia with risk of hepatic encephalopathy. Shunt embolization aims to redirect portal venous flow toward the liver and restore physiologic hepatopetal circulation, with potential clinical benefits including improved hepatic function and regression of associated lesions [31].

**Fig. 5:**
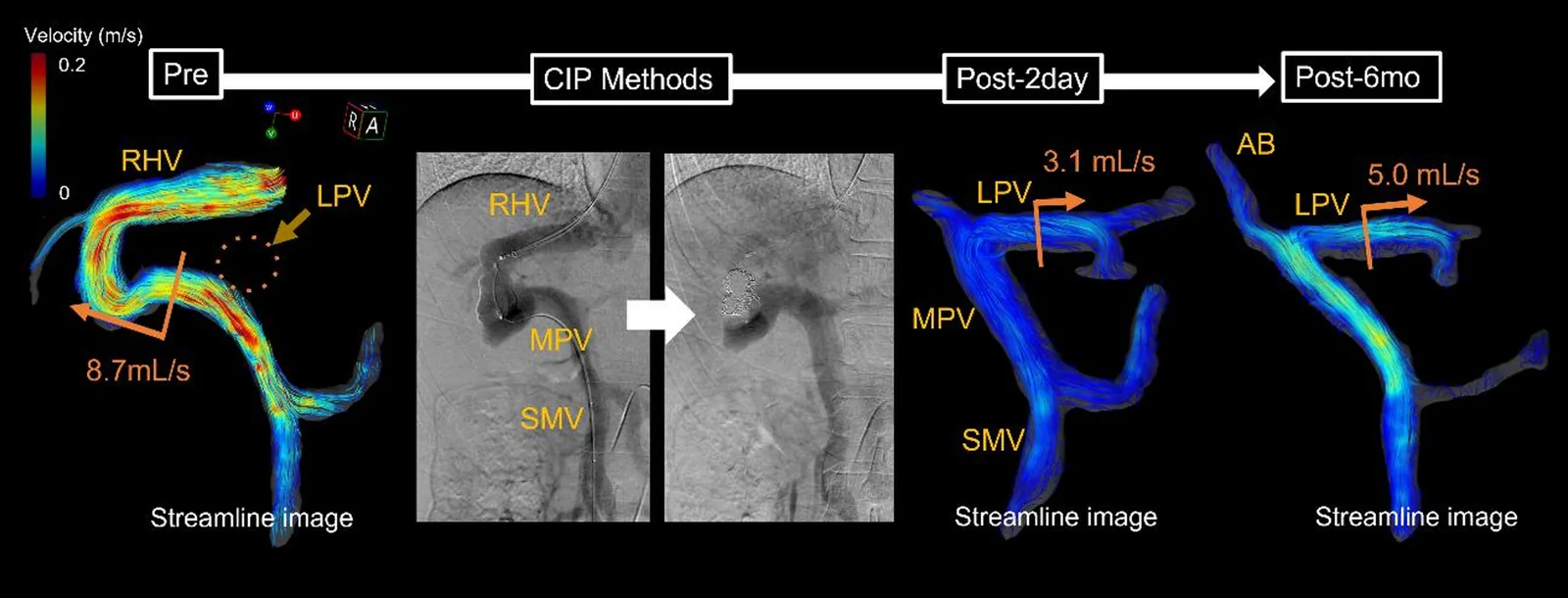
Congenital intrahepatic portosystemic shunt between the posterior portal branch and the right hepatic vein (RHV). Representative case (female, 40 s). Portal flow is largely diverted into the systemic circulation. Transjugular embolization using the coil-in-plug (CIP) technique is performed. Pre-treatment angiography demonstrates a large portosystemic shunt with substantial diversion of portal flow into the systemic circulation. Early post-embolization 4D Flow MRI demonstrates disappearance of shunt flow and redistribution of portal flow into previously underperfused branches, accompanied by transient changes in portal flow velocity. At 6-month follow-up, sustained increase in left portal vein (LPV) and anterior branch (AB) flow is observed, with remodeling of the main portal vein (MPV). These findings demonstrate longitudinal assessment of portal flow redistribution and vascular remodeling using 4D Flow MRI. SMV, superior mesenteric vein. Adapted from Horiguchi et al. Radiology Case Reports 2025, licensed under CC BY-NC-ND 4.0.

Compared with conventional morphologic imaging, 4D Flow MRI enables comprehensive visualization and quantification of portal inflow, shunt outflow, and intrahepatic flow distribution within a single examination (Fig. 5) [32]. This allows direct assessment of the magnitude of shunt flow and the degree of portal flow diversion. Following embolization, 4D Flow MRI can demonstrate reduction or elimination of shunt flow and redistribution of portal perfusion. During follow-up, it may also depict longitudinal remodeling of the portal venous system and associated changes in intrahepatic flow balance, which are difficult to assess noninvasively with conventional imaging alone.

In this context, 4D Flow MRI may provide incremental value in selected patients by supporting physiologic assessment of shunt burden during procedural planning and by enabling noninvasive evaluation of post-embolization hemodynamic remodeling. However, current evidence is limited to case reports and small series, and the relationship between quantified flow parameters and clinical outcomes has not been established. Accordingly, while 4D Flow MRI may aid in physiologic characterization, its role in determining the timing or extent of intervention remains unclear, and routine use in all patients with congenital portal abnormalities is not currently supported.

#### Preoperative portal vein embolization

In patients with advanced hepatobiliary malignancies requiring major hepatectomy, insufficient future liver remnant (FLR) volume or function may result in post-hepatectomy liver failure [33]. To mitigate this risk, preoperative portal vein embolization (PVE) is performed to redirect portal inflow toward the FLR and induce compensatory hypertrophy [33, 34]. In current practice, treatment response is assessed using volumetric CT and functional testing several weeks after the procedure to determine surgical feasibility. In cases of insufficient hypertrophy, adjunctive strategies such as hepatic vein embolization may be considered to further augment FLR growth [35, 36].

**Fig. 6:**
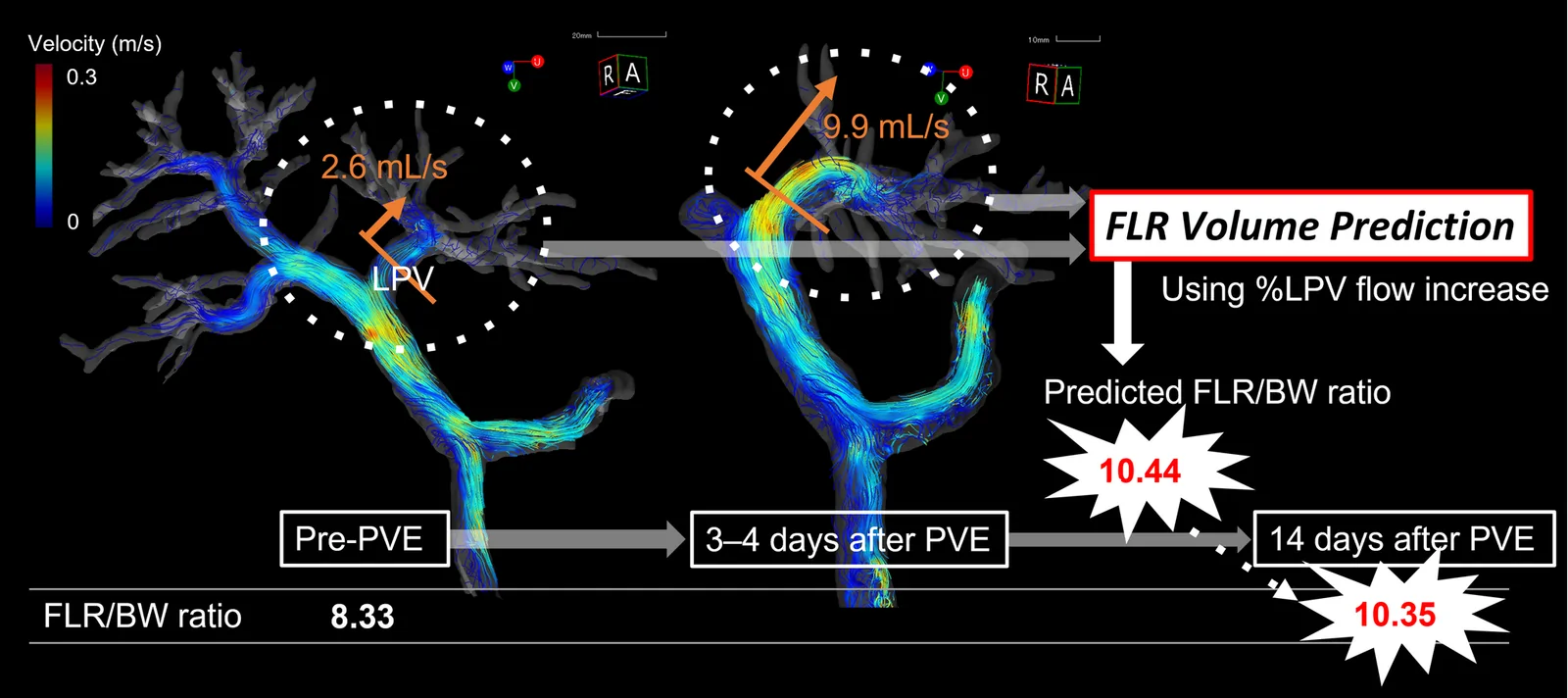
Prediction of future liver remnant (FLR) hypertrophy after preoperative portal vein embolization (PVE). Representative case (female, 70 s). The percentage increase in left portal vein (LPV) flow (%LPV flow increase), calculated from 4D Flow MRI acquired before and 3–4 days after PVE, is used to predict subsequent FLR volumetric gain. In this representative case, the pre-PVE FLR-to-body weight (BW) ratio is 8.33 mL/kg. Based on the early post-PVE increase in LPV flow, the predicted FLR/BW ratio at follow-up is 10.44 mL/kg, closely matching the actual value of 10.35 mL/kg measured 14 days after PVE. These findings illustrate that early changes in portal flow measured by 4D Flow MRI may be associated with subsequent FLR hypertrophy in this cohort. Further validation is required before flow-based prediction can be incorporated into routine clinical decision-making. Adapted from Hyodo R et al. Radiologia Medica 2022, licensed under CC BY 4.0.

4D Flow MRI enables quantitative assessment of early portal hemodynamic changes following PVE, before measurable volumetric hypertrophy occurs. In a prospective study (Fig. 6) [3], 4D Flow MRI performed 3–4 days after right portal vein embolization demonstrated significant increases in left portal vein (LPV) flow, and the percentage increase in LPV flow was strongly correlated with subsequent FLR hypertrophy on follow-up CT (*r* = 0.78). These findings suggest that early hemodynamic response may reflect the capacity for later volumetric adaptation.

Such early physiologic assessment may provide incremental value in identifying patients with insufficient hypertrophic response at an earlier stage, potentially supporting risk stratification or closer monitoring before conventional volumetric assessment becomes available. However, the current evidence is limited to a single-center cohort, and proposed predictive models remain unvalidated and should be considered hypothesis-generating. Accordingly, while early flow-based metrics may provide useful physiologic insight, their role in determining surgical timing or guiding clinical decision-making has not been established, and 4D Flow MRI should be regarded as a complementary investigational tool rather than a replacement for standard assessment.

### Hepatic venous outflow interventions

Hepatic venous outflow disorders represent a distinct hemodynamic environment characterized by venous congestion, collateral formation, and complex multidirectional flow. In these conditions, morphologic imaging alone often underestimates the physiologic consequences of obstruction.

#### IR for Budd-Chiari syndrome

Budd–Chiari syndrome is characterized by venous outflow obstruction from the liver, occurring at the level of the hepatic veins or the inferior vena cava (IVC), and is broadly classified into hepatic vein-type and IVC-type disease [37, 38]. Venous outflow obstruction leads to hepatic congestion, progressive fibrosis, and secondary portal hypertension, often accompanied by extensive collateral formation, resulting in a complex multidirectional hemodynamic environment.

**Fig. 7:**
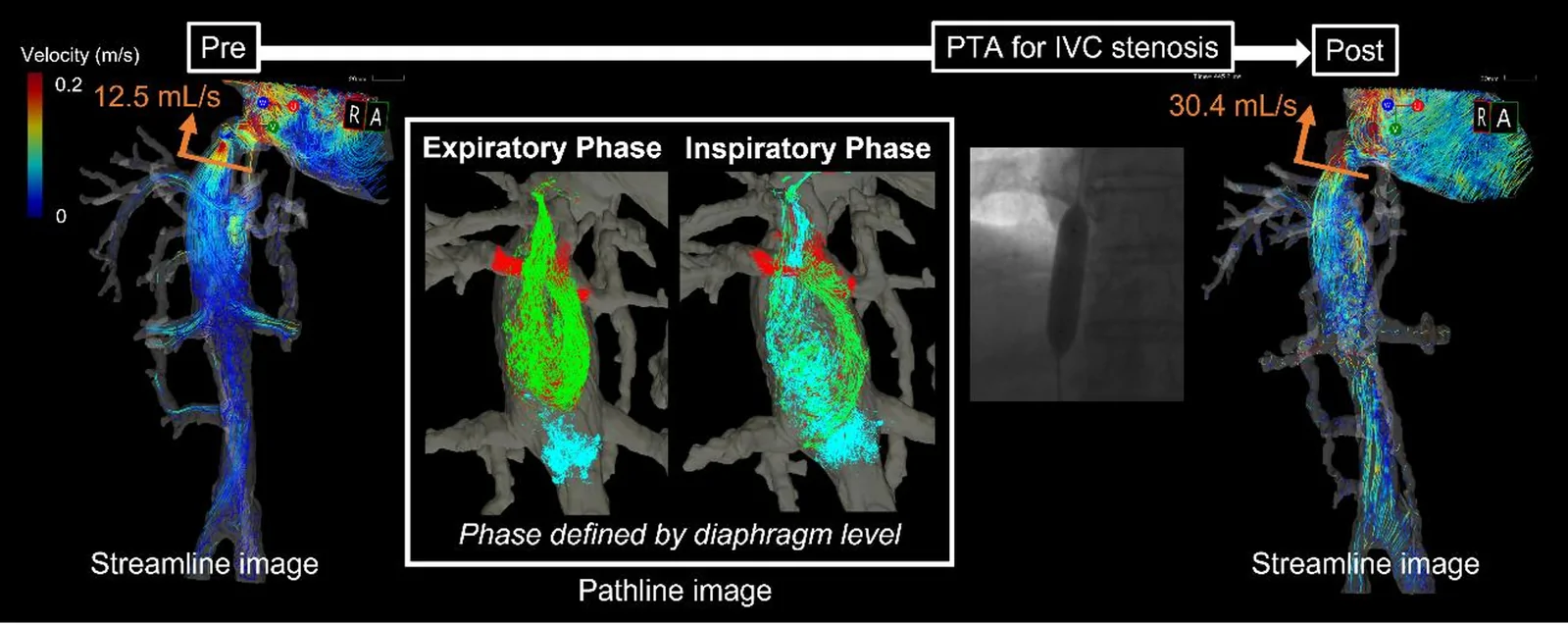
Hemodynamic assessment and treatment response in Budd–Chiari syndrome. Representative case (male, 50 s). The patient has complete occlusion of the three hepatic veins and severe membranous stenosis of the hepatic inferior vena cava (IVC). Respiration-resolved 4D Flow MRI demonstrates phase-dependent hemodynamic changes defined by diaphragm position. Pathline images reveal flow stagnation in the infrarenal IVC during expiration and a high-velocity jet at the site of membranous stenosis. Balloon percutaneous transluminal angioplasty (PTA) is performed. Post-treatment 4D Flow MRI demonstrates increased hepatic IVC flow and global improvement in venous drainage. These findings illustrate comprehensive evaluation of complex venous hemodynamics and treatment response within a single volumetric acquisition. Adapted from Hyodo R et al. Magn Reson Med Sci 2023, licensed under CC BY-NC-ND 4.0.

Compared with conventional morphologic imaging, 4D Flow MRI enables comprehensive assessment of this hemodynamic complexity within a single volumetric acquisition (Fig. 7) [39]. The location and functional severity of venous obstruction, collateral drainage pathways, and portal venous inflow can be simultaneously visualized and quantified. In particular, high-velocity jets at sites of focal stenosis may help identify the hemodynamically dominant lesion, which may be relevant for procedural planning in selected cases. Following endovascular treatment, 4D Flow MRI can demonstrate restoration of flow across the treated segment and improvement in global venous drainage.

In this context, 4D Flow MRI may provide incremental value in selected patients by improving physiologic characterization when morphologic findings alone do not adequately explain clinical severity, or by supporting post-treatment assessment of hemodynamic response. However, current evidence remains limited, and the extent to which these findings should influence procedural strategy or predict outcomes has not been established. Accordingly, 4D Flow MRI is best regarded as a complementary tool in selected cases rather than a determinant of routine clinical decision-making.

#### Transjugular intrahepatic portosystemic shunt (TIPS)

TIPS is a minimally invasive procedure that reduces portal hypertension by creating a covered stent-graft tract between a portal vein branch and a hepatic vein (or the IVC), thereby diverting a portion of portal blood flow into the systemic circulation [40]. It is an established treatment for complications of portal hypertension, particularly variceal bleeding and refractory ascites. Clinical efficacy is primarily assessed by reduction of the portosystemic pressure gradient (PSPG), with values below 12 mmHg associated with reduced rebleeding risk [41–43]. After TIPS placement, both excessive shunting leading to hepatic encephalopathy and insufficient shunt flow due to stenosis or occlusion must be considered. Doppler ultrasound remains the primary surveillance modality, although its diagnostic performance is limited and concordance with invasive findings is imperfect [44].

Compared with Doppler ultrasound, 4D Flow MRI provides comprehensive time-resolved three-dimensional visualization and quantification of flow within the portal venous system and the TIPS tract in a single acquisition [45]. Hemodynamic parameters such as changes in portal flow distribution and the TIPS-to-portal vein flow ratio (shunt fraction) can be quantified [46]. Abnormal velocities or localized turbulence within the shunt have also been reported to correlate with angiographically confirmed stenosis [47]. In addition, computational approaches incorporating 4D Flow-derived data have demonstrated the feasibility of noninvasive estimation of PSPG [48].

In this context, 4D Flow MRI may provide incremental value in selected patients with suspected TIPS dysfunction by enabling comprehensive physiologic assessment and helping identify cases in which further invasive evaluation is warranted. However, current evidence remains limited, and its role relative to Doppler ultrasound in routine surveillance has not been established. Accordingly, 4D Flow MRI is best considered a complementary, problem-solving tool for triaging patients rather than a replacement for Doppler ultrasound or invasive pressure measurement.

## Technical considerations for clinical interpretation in abdominal IR

Although 4D Flow MRI provides comprehensive visualization and quantification of vascular hemodynamics, accurate clinical interpretation in abdominal IR requires careful attention to acquisition strategy, physiologic variability, and post-processing methodology. Without appropriate technical awareness, misinterpretation of flow patterns or quantitative parameters may occur [1, 4, 5]. In clinical practice, these technical factors directly influence the reliability of hemodynamic interpretation and the extent to which 4D Flow MRI findings can support decision-making.

### Velocity encoding and dynamic range

Appropriate VENC selection is fundamental. Abdominal vascular territories often contain both high-velocity components (e.g., normal aortic site, portal vein or IVC stenosis, TIPS tract) and low-velocity components (e.g., type II endoleak inflow, portal venous redistribution, intra-aneurysmal recirculation). A single VENC setting may therefore be suboptimal. Excessively low VENC results in phase aliasing at stenotic segments, whereas high VENC reduces velocity-to-noise ratio and compromises quantification of slow flow [4, 5, 14, 15]. Dual- or multi-VENC strategies may improve dynamic range, although at the cost of increased complexity and longer acquisition times. These approaches are particularly useful when both high-velocity aortic flow and low-velocity components must be assessed simultaneously, such as after EVAR. In practice, appropriate VENC planning remains an important determinant of whether a study will be clinically interpretable. In abdominal applications, relatively slow flow velocities and large body habitus may further complicate acquisition, requiring optimization of signal-to-noise ratio and appropriate use of fat suppression or inner volume excitation.

### Physiologic variability in portal hemodynamics

Portal venous flow is influenced not only by technical acquisition parameters but also by physiologic conditions [7, 49]. While respiratory phase may affect flow magnitude and direction, most clinical protocols standardize acquisition at end-expiration to reduce variability. More importantly, portal hemodynamics are sensitive to prandial state and time of examination. Postprandial increases in SMV and MPV flow have been demonstrated, with variations depending on nutrient composition, and may alter both the magnitude and direction of portosystemic shunt flow. Diurnal variation has also been reported, with higher portal flow observed later in the day compared with morning measurements. Accordingly, fasting status, dietary timing, and examination time should be considered. These factors should be kept consistent when serial quantitative assessment is performed, particularly in portal venous interventions.

### Metal implants and disturbed flow

Stent grafts and venous stents introduce susceptibility artifacts and intravoxel dephasing, potentially leading to underestimation of peak velocities or signal voids [4, 5]. Turbulent or transitional flow distal to stenosis may further degrade quantitative reliability. Although shortened echo times and optimized spatial resolution may help reduce metal-related artifacts, quantitative reliability within stented segments remains limited, necessitating careful segment selection. Quantification should preferentially be performed in segments demonstrating organized axial flow, and correlation with morphologic imaging remains essential.

### Quantitative reproducibility

Flow measurements depend on accurate vessel segmentation, plane orientation, and background phase correction [4, 5, 7, 50]. Segmentation of vascular structures from magnitude or 3D phase-contrast images may influence measurement reproducibility, particularly in small vessels or regions with low signal intensity. In such cases, higher spatial resolution may be required, although this may increase acquisition time and limit clinical feasibility. Oblique plane placement or partial-volume effects may significantly alter calculated flow rates. Internal consistency checks, such as conservation of mass at vascular bifurcations (e.g., MPV = SMV + SV = LPV + RPV), may support data plausibility. Standardized reporting of acquisition and analysis parameters is necessary to improve reproducibility and to facilitate more consistent implementation across centers.

### Interpretation of advanced metrics

4D Flow-derived parameters, including wall shear stress (WSS), oscillatory shear index (OSI), and EL, are theoretically attractive because they may capture aspects of vascular hemodynamics not reflected by conventional morphologic imaging; however, their current clinical interpretability remains limited [4, 5, 10]. These metrics are highly sensitive to spatial resolution, segmentation strategy, and post-processing methodology, and absolute thresholds or reference ranges have not been established. Variability in acquisition and analysis techniques further limits reproducibility across institutions. In addition, the spatial resolution of current 4D Flow MRI is insufficient for accurate characterization of near-wall velocity gradients, and therefore WSS-derived parameters should be interpreted as approximate rather than true physiologic quantities. As a result, direct comparison between patients is challenging, and these metrics are more appropriately interpreted in a relative manner within the same patient, such as in pre- and post-intervention assessments. In contrast, flow velocity and volumetric flow rate are currently more robust and reproducible parameters and remain the most clinically interpretable quantitative measures derived from 4D Flow MRI. In practical terms, advanced hemodynamic metrics such as WSS, OSI, and EL should be regarded as adjunctive or research-oriented parameters rather than clinically actionable metrics, and their role in routine clinical decision-making remains limited.

### Barriers to widespread clinical implementation

Despite growing interest, several barriers limit routine clinical adoption. Acquisition times remain longer than conventional imaging, potentially limiting feasibility in patients with unstable or irregular respiration. Although accelerated imaging techniques have improved practicality [51, 52], workflow integration remains suboptimal in many institutions. Post-processing remains time-consuming and requires specialized expertise and dedicated software. Variability in vessel segmentation, plane selection, and interpretation of complex multidirectional flow patterns may limit reproducibility [50]. Furthermore, standardized acquisition protocols and reference values for advanced hemodynamic parameters are lacking, restricting consistent application across centers.

These technical and workflow-related constraints may be particularly relevant outside tertiary referral centers or research-oriented institutions with dedicated expertise in advanced flow imaging. At present, clinical implementation is likely to be most realistic in academic centers, high-volume referral practices, and selected complex cases in which morphology-based imaging alone does not adequately answer the relevant physiologic question. Broader adoption will likely depend on further workflow simplification, automation of segmentation and quantification, and validation of clinically actionable hemodynamic metrics.

### Clinical workflow and practical considerations

From a practical perspective, the use of 4D Flow MRI in abdominal IR should be guided by whether additional hemodynamic information is expected to influence clinical interpretation beyond morphology alone. In routine clinical practice, CT, Doppler ultrasound, and conventional angiography remain the primary imaging modalities because of their availability, speed, and established roles in diagnosis and treatment planning. 4D Flow MRI is most appropriately used in selected cases where morphology-based imaging is insufficient to explain physiologically relevant findings, such as complex flow redistribution, unclear functional significance of vascular lesions, or discordance between imaging findings and clinical presentation. In such scenarios, it may serve as a complementary or problem-solving tool rather than a first-line modality. Conversely, when morphologic imaging adequately defines disease severity and treatment strategy, the additional information provided by 4D Flow MRI is unlikely to alter management and may not justify the additional acquisition time, cost, and post-processing effort. This is particularly relevant in routine surveillance settings, such as standard post-EVAR follow-up or TIPS monitoring, where established modalities such as CT or Doppler ultrasound are generally sufficient. At present, practical limitations such as longer acquisition times, limited availability, and the need for specialized expertise restrict widespread implementation. Accordingly, integration of 4D Flow MRI into clinical workflows should be selective and question-driven, focusing on cases in which physiologic assessment is expected to provide incremental value beyond conventional imaging.

## Current evidence landscape

Clinical evidence supporting abdominal 4D Flow MRI in IR remains heterogeneous and is derived predominantly from observational studies. Most published data consist of single-center prospective or retrospective cohorts, case series, and technical feasibility studies, with substantial variation in patient populations, vascular territories, acquisition protocols, and quantitative endpoints (Table 1). Although recurring hemodynamic patterns have been reported across different applications, randomized studies and large multicenter outcome-driven investigations are currently lacking.

**Table 1:** Summary of current clinical evidence for abdominal 4D Flow MRI in interventional radiology

Clinical application	Study / *N*	Main finding	Current evidence status
EVAR hemodynamics	Horiguchi et al., JMRI 2023 [2]; observational cohort, *N* = 13	Local energy loss increased after EVAR and was higher in cases with stent-graft limb deformity or focal stenosis.	Preliminary physiologic relevance
Endoleak characterization	Sakata et al., Circ J 2016 [13]; observational study, *N* = 31	4D Flow MRI detected endoleaks more sensitively than CTA, differentiated concomitant endoleaks, and subclassified type II endoleaks based on flow pattern.	Pilot observational evidence with adjunctive diagnostic value
BRTO / portosystemic shunt embolization	Hyodo et al., Hepatol Res 2021 [22]; observational study, *N* = 2	4D Flow MRI quantified shunt severity and demonstrated restoration of hepatopetal portal flow after embolization.	Feasibility with clinical physiologic relevance
Portal vein stenting	Hyodo et al., MRMS 2021 [29]; case-based observational study, *N* = 1	4D Flow MRI depicted hemodynamic improvement after portal vein stenting, including more homogeneous intrahepatic portal perfusion and reduced collateral flow.	Feasibility in selected cases
Congenital portosystemic shunt embolization	Horiguchi et al., Radiol Case Rep 2025 [32]; case report, *N* = 1	4D Flow MRI quantified shunt burden and documented post-embolization hemodynamic remodeling during follow-up.	Case-based evidence only
Preoperative portal vein embolization	Hyodo et al., Radiology 2023 [3]; prospective cohort, *N* = 37	Early increase in left portal vein flow after PVE was associated with subsequent future liver remnant hypertrophy.	Promising but not externally validated
Budd–Chiari syndrome	Hyodo et al., MRMS 2023 [39]; case-based feasibility study, *N* = 1	4D Flow MRI depicted obstruction severity, collateral venous drainage, and post-treatment hemodynamic change.	Feasibility only
TIPS hemodynamic change after placement	Stankovic et al., Eur Radiol 2015 [45]; prospective observational study, *N* = 11	4D Flow MRI demonstrated post-TIPS changes in portal and splanchnic arterial flow and identified shunt stenosis in two patients.	Prospective feasibility study with physiologic monitoring value
TIPS hemodynamic monitoring	Bannas et al., Radiology 2016 [46]; prospective observational study, *N* = 7	4D Flow MRI enabled longitudinal monitoring of post-TIPS hepatic blood flow and suggested that the TIPS-to-portal vein flow ratio may reflect shunt adequacy.	Prospective observational physiologic monitoring
TIPS dysfunction assessment	Owen et al., JVIR 2018 [47]; feasibility/correlation study, *N* = 23 enrolled, 16 analyzable	Abnormal velocities and focal turbulence on 4D Flow MRI identified venography-confirmed TIPS stenosis in technically successful cases.	Feasibility study with preliminary adjunctive value
TIPS pressure estimation	Riedel et al., Radiology 2024 [48]; correlation study, *N* = 20	4D Flow MRI-based CFD enabled noninvasive PSPG estimation with strong correlation to invasive measurements and 80% classification accuracy for PSPG > 12 mm Hg.	Prospective feasibility/correlation study; investigational but promising

The strength of evidence also varies by clinical application. In aortic interventions, the literature focuses primarily on feasibility, flow visualization, endoleak characterization, and energy-related parameters in comparison with CT or angiography. In portal venous interventions, evidence includes prospective cohort studies evaluating early hemodynamic change and its association with subsequent morphologic or volumetric outcomes, but external validation remains limited. In hepatic venous outflow disorders and TIPS evaluation, the available literature consists largely of feasibility studies and correlation analyses with invasive angiographic or pressure-based measurements.

Accordingly, current evidence supports the physiologic plausibility and technical promise of 4D Flow MRI across several abdominal IR applications; however, important limitations remain. Much of the literature is based on small, single-center cohorts and is therefore susceptible to selection bias. In addition, considerable heterogeneity in acquisition protocols, analysis methods, and reported endpoints limits reproducibility across institutions and complicates direct comparison between studies. Perhaps more importantly, outcome-based validation remains scarce, and the relationship between observed hemodynamic parameters and clinically meaningful endpoints has not been consistently established. Taken together, these factors continue to constrain clinical adoption. In practical terms, the current evidence base supports the use of 4D Flow MRI primarily as a complementary or problem-solving tool in selected cases where morphology alone is insufficient, while its role in routine clinical decision-making remains limited. At present, the literature is better suited to defining promising use cases and research priorities than to supporting routine clinical implementation.

## Future prospects

Several technological and methodological developments are likely to improve the practicality of 4D Flow MRI in abdominal IR. In the near term, accelerated acquisition techniques, including compressed sensing and parallel imaging, may further reduce scan times while maintaining clinically acceptable image quality, thereby improving feasibility in routine practice [51, 52]. More recently, deep learning-based reconstruction approaches have been explored to support highly accelerated acquisitions through undersampling and improve reconstruction performance [53]. In parallel, artificial intelligence-based automated segmentation and flow analysis are expected to reduce post-processing time, improve reproducibility, and facilitate more streamlined clinical workflows [54–58]. These advances may help lower current barriers related to acquisition burden, operator dependence, and analysis time.

Equally important, multicenter collaborative studies will be needed to establish harmonized acquisition protocols, validate hemodynamic biomarkers, and determine in which clinical scenarios 4D Flow MRI provides meaningful incremental value beyond conventional imaging [5]. Such efforts will be essential for moving the field from promising single-center experience toward broader clinical applicability.

Over the longer term, integration with CFD-based modeling and noninvasive estimation of pressure gradients derived from flow data may allow more comprehensive, patient-specific physiologic assessment without the need for invasive measurements [48]. If technical simplification and prospective validation can be achieved, these approaches may expand the role of 4D Flow MRI beyond descriptive hemodynamic assessment. However, such applications remain dependent on overcoming current limitations in standardization, reproducibility, and clinical validation.

As IR increasingly incorporates precision-oriented decision-making, quantitative hemodynamic imaging may contribute to a broader shift from purely morphologic evaluation toward more physiology-informed assessment. However, its integration into routine clinical decision-making remains to be established.

## Conclusion

4D Flow MRI enables comprehensive, noninvasive, and quantitative assessment of vascular hemodynamics that complements conventional imaging approaches in abdominal IR. Its greatest potential lies in clinical scenarios in which morphology-based imaging alone does not sufficiently characterize physiologically relevant flow abnormalities, treatment-related hemodynamic change, or functional disease severity. Across aortic, portal venous, and hepatic venous interventions, 4D Flow MRI provides unique insight into multidirectional flow, collateral pathways, and post-interventional physiologic response. However, important technical, methodological, and evidentiary challenges remain, including workflow integration, reproducibility, and limited outcome-based validation. In practical terms, current evidence supports the use of 4D Flow MRI primarily as a complementary or problem-solving tool in selected cases rather than as a modality for routine clinical decision-making. Continued advances in acquisition strategies, automated analysis, and multicenter validation will be required to define where it provides meaningful incremental clinical value. With further validation, 4D Flow MRI may contribute to a more physiology-informed approach to IR practice, although its integration into routine clinical workflows remains to be established.

## Ethics statement

Representative cases and imaging data included in this review were derived from institutional datasets. The study was conducted in accordance with institutional ethical standards, and informed consent for research use of imaging data was obtained from all patients where applicable.

## Data Availability

No datasets were generated or analysed during the current study.
